# Association of patients’ sex with treatment outcomes after intravesical bacillus Calmette–Guérin immunotherapy for T1G3/HG bladder cancer

**DOI:** 10.1007/s00345-021-03653-1

**Published:** 2021-03-13

**Authors:** David D’Andrea, Francesco Soria, Anne J. Grotenhuis, Eugene K. Cha, Nuria Malats, Savino Di Stasi, Steven Joniau, Tommaso Cai, Bas W. G. van Rhijn, Jaques Irani, Jeffrey Karnes, John Varkarakis, Jack Baniel, Joan Palou, Marek Babjuk, Martin Spahn, Peter Ardelt, Renzo Colombo, Vincenzo Serretta, Guido Dalbagni, Paolo Gontero, Riccardo Bartoletti, Stephane Larré, Per-Uno Malmstrom, Richard Sylvester, Shahrokh F. Shariat

**Affiliations:** 1 Department of Urology, Comprehensive Cancer Center, Medical University of Vienna, Vienna, Austria; 2 Division of Urology, University of the Studies of Turin, AOU Città Della Salute e Della Scienza di Torino, Presidio Molinette, Turin, Italy; 3 Department of Urology, Radboud University Nijmegen Medical Centre, Nijmegen, The Netherlands; 4 Department of Urology, Weill Medical College of Cornell, University, New York, NY USA; 5 Department of Urology, Memorial Sloan Kettering Cancer Center, New York, NY USA; 6 Genetic and Molecular Epidemiology Group, Spanish National Cancer Research Centre (CNIO), Madrid, Spain; 7 Department of Urology, Policlinico Tor Vergata-University of Rome, Rome, Italy; 8 Oncologic and Reconstructive Urology, Department of Urology, University Hospitals Leuven, Leuven, Belgium; 9 Department of Urology, Santa Chiara Hospital, Trento, Italy; 10 Department of Surgical Oncology (Urology), Netherlands Cancer Institute-Antoni Van Leeuwenhoek Hospital, Amsterdam, The Netherlands; 11 Department of Urology, Centre Hospitalier Universitaire La Milétrie, University of Poitiers, Poitiers, France; 12 Department of Urology, Mayo Clinic, Rochester, MN USA; 13 Department of Urology, Sismanoglio Hospital, University of Athens, Athens, Greece; 14 Department of Urology, Rabin Medical Centre, Tel Aviv, Israel; 15 Department of Urology, Fundacio Puigvert, University of Barcelona, Barcelona, Spain; 16 Department of Urology, Motol Hospital, University of Praha, Prague, Czech Republic; 17 Department of Urology, University Hospital of Wuerzburg, Wuertzburg, Germany; 18 Department of Urology, Universitätsklinik Freiburg, Freiburg, Germany; 19 Departement of Urology, Università Vita Salute, Ospedale S. Raffaele, Milan, Italy; 20 Department of Surgical, Oncological and Stomatological Sciences, University of Palermo, Palermo, Italy; 21 Department of Translational Research and New Technologies, University of Pisa, Pisa, Italy; 22 Department of Urology, CHU de Reims, Reims, France; 23 Department of Urology, Academic Hospital, Uppsala University, Uppsala, Sweden; 24 European Association of Urology Non-Muscle Invasive Bladder Cancer Guidelines Panel, Brussels, Belgium; 25 Department of Urology, Second Faculty of Medicine, Charles University, Prague, Czech Republic; 26 Institute for Urology and Reproductive Health, I.M. Sechenov First Moscow State Medical University, Moscow, Russia; 27 Department of Urology, University of Jordan, Amman, Jordan; 28 European Association of Urology Research Foundation, Arnhem, The Netherlands

**Keywords:** Bladder cancer, BCG, Response, Age, Progression, Recurrence

## Abstract

**Purpose:**

To investigate the association of patients’ sex with recurrence and disease progression in patients treated with intravesical bacillus Calmette–Guérin (BCG) for T1G3/HG urinary bladder cancer (UBC).

**Materials and methods:**

We analyzed the data of 2635 patients treated with adjuvant intravesical BCG for T1 UBC between 1984 and 2019. We accounted for missing data using multiple imputations and adjusted for covariate imbalance between males and females using inverse probability weighting (IPW). Crude and IPW-adjusted Cox regression analyses were used to estimate the hazard ratios (HR) with their 95% confidence intervals (CI) for the association of patients’ sex with HG-recurrence and disease progression.

**Results:**

A total of 2170 (82%) males and 465 (18%) females were available for analysis. Overall, 1090 (50%) males and 244 (52%) females experienced recurrence, and 391 (18%) males and 104 (22%) females experienced disease progression. On IPW-adjusted Cox regression analyses, female sex was associated with disease progression (HR 1.25, 95%CI 1.01–1.56, *p* = 0.04) but not with recurrence (HR 1.06, 95%CI 0.92–1.22, *p* = 0.41). A total of 1056 patients were treated with adequate BCG. In these patients, on IPW-adjusted Cox regression analyses, patients’ sex was not associated with recurrence (HR 0.99, 95%CI 0.80–1.24, *p* = 0.96), HG-recurrence (HR 1.00, 95%CI 0.78–1.29, *p* = 0.99) or disease progression (HR 1.12, 95%CI 0.78–1.60, *p* = 0.55).

**Conclusion:**

Our analysis generates the hypothesis of a differential response to BCG between males and females if not adequately treated. Further studies should focus on sex-based differences in innate and adaptive immune system and their association with BCG response.

**Supplementary Information:**

The online version contains supplementary material available at 10.1007/s00345-021-03653-1.

## Introduction

Standard treatment of high-risk non-muscle-invasive bladder cancer (NMIBC) is complete transurethral resection (TURB) followed by adjuvant intravesical immunotherapy with bacillus Calmette–Guérin (BCG) [1, 2]. Despite adequate treatment, around 40% of patients with T1 urinary bladder cancer (UBC) will experience disease recurrence and around 20% disease progression, respectively [2–4]. Identifying the patients who are most likely to recur or progress during or after adjuvant BCG would be highly valuable in the clinical decision-making process and patient counselling. These patients could be offered alternative treatment strategies such as early/immediate radical cystectomy [3]. To address this unmet need, an effort has been put into the development of prognostic models and risk stratification tools such as those from the European Organization for Research and Treatment of Cancer (EORTC) and the Club Urológico Español de Tratamiento Oncológico (CUETO) [5, 6]. However, the performance of these models is still under debate, in part because they do not take in account the heterogeneous genomic landscape and mutational burden of the disease [7], or other adverse pathologic features such as lympho-vascular invasion and variant histology [8–12]. Moreover, sex-based differences in biology, epidemiology, and outcomes of UBC are well documented in muscle-invasive UBC [13, 14]. However, the association of patients’ sex with treatment outcome to BCG therapy is still controversial [5, 7, 15, 16] and has so far been implemented only in the CUETO risk tables [5].

To fill this gap in knowledge, we investigated the impact of sex on clinical outcomes of patients treated with TURB and BCG for T1 G3/high-grade (HG) UBC.

## Methods

### Study population

We analyzed the data of 2635 patients treated with adjuvant intravesical BCG for T1 UBC between 1984 and 2019 within a multicenter collaboration including 25 centers across Europe and the United States of America.

### TURB and adjuvant BCG instillations

A second look TURB was performed at the physician’s discretion based on pathologic and intraoperative findings. All surgical specimens were processed according to standard pathologic procedures and staged according to the TNM classification. Tumor grade was assigned according to the 1973 and/or 2004 World Health Organization system.

Metastatic disease and concomitant upper urinary-tract urothelial carcinoma were excluded using radiological imaging.

Due to the retrospective nature of the study, the indication and duration for adjuvant BCG therapy were given at the physician's discretion. An adequate BCG treatment was defined as the administration of at least five of six doses of an initial induction course plus at least two of three doses of maintenance therapy or the administration of at least five of six doses of an initial induction course plus at least two of six doses of a second induction course [17].

### Follow-up

Due to the retrospective nature of the study, follow-up was not standardized but rather performed according to institutional standards and based on guidelines at the time and at the physician’s discretion.

### Outcome measurement

The primary endpoint was the association of patients’ sex with disease recurrence, during BCG therapy or after completion of BCG therapy. The secondary endpoint was the association of patients’ sex with disease progression to MIBC.

The “time to event” was calculated as the time between first pT1G3/HG diagnosis and a histopathological confirmed recurrence or progression with TURB. Patients who did not develop a recurrence during the follow-up were censored at their last follow-up visit.

### Statistical analyses

Statistical analyses were performed in different steps. First, to account for missing baseline data that were assumed to be missing at random, we performed multiple imputations by using chained equations. Thirty imputed data sets were generated using predictive mean matching for numeric variables, logistic regression for binary variables, and Bayesian polytomous regression for factor variables. Second, we used inverse probability weighting (IPW) to reduce the bias of unweighted estimators and adjust for covariates imbalance between males and females. The variables used to estimate the inverse probability weights were age, smoking status, exposure to chemical compounds, previous low-grade UBC, previous intravesical therapy, presence of detrusor muscle in the TURB specimen, G3 grade, multifocal T1 UBC, tumor size > 3 cm, concomitant CIS, second look TURB, number of BCG induction cycles and number of BCG maintenance cycles. Post-weighting balance in covariates was evaluated by using standardized mean differences (supplementary figure S1). Third, unweighted and IPW-adjusted univariable Cox proportional hazard regression analyses were used to estimate the hazard ratios (HR) with their 95% confidence intervals (CI) for the association of patients’ sex with recurrence and disease progression. Fourth, we estimated recurrence and disease progression rates between groups using cumulative incidence curves.

Finally, we performed subgroups analyses in the weighted population using IPW-adjusted Cox proportional hazard regression analyses investigating the association of sex with disease recurrence, HG-recurrence, and disease progression in patients adequately treated with BCG. Outcome estimates were graphically visualized using cumulative incidence curves.

Statistical significance was considered at *p* < 0.05. All tests were performed with R (R Foundation for Statistical Computing, v3.5.1).

## Results

A total of 2170 (82%) males and 465 (18%) females were available for analysis. Unweighted and weighted clinicopathologic features of the patients, stratified by sex, are shown in Table 1. Standardized differences of unweighted comparisons showed that both groups differed significantly with respect to age, smoking status, exposure to chemical compounds and detrusor muscle in the TURB specimen. Overall, 64% of patients without muscle at first TURB underwent a second look TURB and 37% of patients with muscle at first TURB underwent a second look TURB.

**Table 1:** Baseline characteristics of 2635 patients treated with transurethral resection of the bladder (TURB) and adjuvant intravesical bacillus Calmette–Guérin (BCG) for pT1G3/HG urinary bladder cancer (UBC) in unweighted and weighted study population after multiple imputation

	Unweighted				Weighted			
	Male	Female	*p*	SMD	Male	Female	*p*	SMD
*n*	2170	465			2634.6	2638.8		
Age, median (IQR)	68 (60–74)	69 (61–76)	< 0.01	0.11	68 (61–74)	68 (59–75)	0.56	0.08
Smoking status, *n* (%)								
Never	523 (24.1)	244 (52.5)	< 0.01	0.62	766.5 (29.1)	766.2 (29.0)	0.95	0.02
Former	955 (44.0)	115 (24.7)			1070.0 (40.6)	1091.6 (41.4)		
Current	692 (31.9)	106 (22.8)			798.1 (30.3)	781.1 (29.6)		
Exposure to chemical compounds, *n* (%)								
No	1977 (91.1)	453 (97.4)	< 0.01	0.27	2429.6 (92.2)	2443.7 (92.6)	0.87	0.01
Yes	193 (8.9)	12 (2.6)			204.9 (7.8)	195.2 (7.4)		
Previous LG UBC, *n* (%)								
No	1928 (88.8)	416 (89.5)	0.76	0.02	2343.8 (89.0)	2347.1 (88.9)	0.99	< 0.01
Yes	242 (11.2)	49 (10.5)			290.7 (11.0)	291.7 (11.1)		
Previous intravesical therapy, *n* (%)								
No	2053 (94.6)	445 (95.7)	0.40	0.05	2497.2 (94.8)	2495.6 (94.6)	0.88	0.01
Yes	117 (5.4)	20 (4.3)			137.3 (5.2)	143.2 (5.4)		
Detrusor muscle in the TURB specimen, *n* (%)								
No	369 (17.0)	99 (21.3)	0.03	0.11	466.1 (17.7)	455.4 (17.3)	0.84	0.01
Yes	1801 (83.0)	366 (78.7)			2168.5 (82.3)	2183.5 (82.7)		
Grade G3 (WHO 1973), *n* (%)								
No	134 (6.2)	32 (6.9)	0.64	0.03	167.8 (6.4)	208.5 (7.9)	0.34	0.06
Yes	2036 (93.8)	433 (93.1)			2466.8 (93.6)	2430.3 (92.1)		
High grade (WHO 2004), *n* (%)	1586 (100.0)	354 (100.0)	NA	NA	1938.1 (100.0)	1996.6 (100.0)	NA	NA
Multifocal pT1 UBC, *n* (%)								
No	1425 (65.7)	305 (65.6)	1.00	< 0.01	1731.4 (65.7)	1765.0 (66.9)	0.67	0.02
Yes	745 (34.3)	160 (34.4)			903.2 (34.3)	873.9 (33.1)		
Tumor size > 3 cm, *n* (%)								
No	1439 (66.3)	296 (63.7)	0.30	0.06	1734.5 (65.8)	1717.2 (65.1)	0.79	0.02
Yes	731 (33.7)	169 (36.3)			900.1 (34.2)	921.6 (34.9)		
Concomitant CIS, *n* (%)								
No	1602 (73.8)	358 (77.0)	0.17	0.07	1960.3 (74.4)	1964.1 (74.4)	0.99	< 0.01
Yes	568 (26.2)	107 (23.0)			674.3 (25.6)	674.7 (25.6)		
Invasion of the prostatic urethra, *n* (%)								
No	2058 (94.8)	–	NA	NA	2501.1 (94.9)	–	NA	NA
Yes	112 (5.2)	–			133.5 (5.1)	–		
Second look TURB, *n* (%)								
No	1272 (58.6)	258 (55.5)	0.23	0.06	1532.1 (58.2)	1562.2 (59.2)	0.72	0.02
Yes	898 (41.4)	207 (44.5)			1102.5 (41.8)	1076.6 (40.8)		
Induction BCG instillations, median (IQR)	6 (6–6)	6 (6–6)	0.05	0.10	6 (6–6)	6 (6–6)	0.43	0.01
Maintenance BCG instillations, median (IQR)	9 (5–11)	9 (6–10)	0.62	0.02	9 (5–10)	9 (6–10)	0.72	< 0.01

*IQR*  interquartile range, *LG*  low-grade, *CIS*  carcinoma in situ, *SMD*  standardized mean difference

After IPW adjustment, all relevant standardized differences were less than 10%, which indicated that clinico-pathologic features between groups were subsequently comparable.

**Fig. 1:**
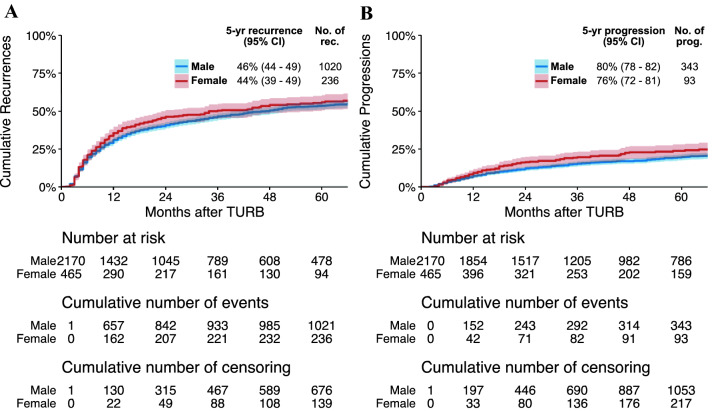
Cumulative incidence curves for the association of patients’ sex with time to disease recurrence (**a**) and progression in 2635 patients treated with transurethral resection of the bladder (TURB) and adjuvant intravesical bacillus Calmette–Guérin (BCG) for T1G3/HG urinary bladder cancer

The median follow-up for alive patients in the cohort was 50 months (IQR 26–88). Within this period, 1090 (50%) males and 244 (52%) females experienced a recurrence, and 391 (18%) males and 104 (22%) females experienced a disease progression. On unweighted univariable Cox regression analyses, female sex was associated with disease progression (crude HR 1.24, 95%CI 1.001–1.54, *p* = 0.04) but not with recurrence (crude HR 1.07, 95%CI 0.93–1.23, *p* = 0.34). On IPW-adjusted univariable Cox regression analyses, female sex was also associated with disease progression (HR 1.25, 95%CI 1.01–1.56, *p* = 0.04) but not with recurrence (HR 1.06, 95%CI 0.92–1.22, *p* = 0.41) (Fig. 1).

Overall, 544 patients, 436 (20%) males and 108 (23%) females, were treated with radical cystectomy for BCG failure. Data on pathologic T-stage were available for 506 patients. Within this group, 146 (36%) males and 34 (34%) females had non-organ confined disease (pT3/pT4 and/or positive nodal stage, *p* = 0.65).

**Fig. 2:**
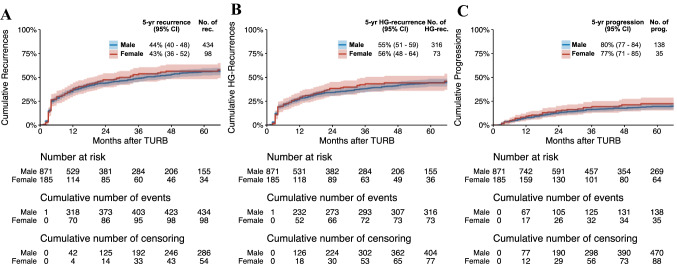
Cumulative incidence curves for the association of patients’ sex with time to disease recurrence (**a**) high-grade (HG) recurrence (**b**) and disease progression (**c**) in 1056 patients treated with transurethral resection of the bladder (TURB) and adequate adjuvant intravesical bacillus Calmette–Guérin (BCG) for T1G3/HG urinary bladder cancer

A total of 1056 patients, 871/2170 males (40%) and 185/465 females (40%), were treated with adequate BCG. Of these, 52% (454) of the males and 54% (99) of the females had recurrences, 38% (328) of the males and 40% (74) of the females had HG-recurrence and 17% (151) of the males and 20% (37) of the females had disease progression. On unweighted univariable Cox regression analyses, patients’ sex was not associated with disease recurrence (crude HR 1.01, 95%CI 0.81–1.26, *p* = 0.91), HG-recurrence (crude HR 1.02, 95%CI 0.79–1.31, *p* = 0.87) or disease progression (crude HR 1.10, 95%CI 0.77–1.58, *p* = 0.60). On IPW-adjusted Cox regression analyses, patients’ sex was not associated with disease recurrence (HR 0.99, 95%CI 0.80–1.24, *p* = 0.96), HG-recurrence (HR 1.00, 95%CI 0.78–1.29, *p* = 0.99) or disease progression (HR 1.12, 95%CI 0.78–1.60, *p* = 0.55) (Fig. 2).

## Discussion

In a retrospective analysis of a large multicenter international dataset of patients treated with TURB and adjuvant intravesical BCG for T1G3/HG UBC, we found an association of female sex with disease progression. However, this difference disappeared in patients adequately treated with BCG.

The difference in incidence and oncologic outcomes of UBC has been widely reported in the literature^14^. These dissimilarities have been partially explained by the activity of the sex steroid hormone pathways, differences in the quality of treatment, and sex-specific differences in immunity [18–21]. However, results across studies are controversial and are mainly related to muscle-invasive UBC.

In NMIBC, the bulk of the evidence presented by the current literature questions the prognostic value and differential response to BCG between males and females.

The CUETO and the 2016 updated EORTC risk tables are the two most used prognostic tools in clinical decision making for patients with UCB treated with BCG [5, 6]. Because patients’ sex was found to be associated with disease recurrence in the CUETO data, sex has been implemented in their model. Neither the CUETO nor the EORTC studies found an association between patient’s sex and disease progression.

The association of patients’ sex with oncologic outcomes of T1 UBC has been investigated in several other retrospectives series, with similar results.

In a retrospective series of 146 patients and in a subgroup analysis of 234 out of 916 patients treated with TURB and adjuvant intravesical BCG, female sex was not found to be an independent predictor of disease recurrence and progression [15, 16].

There are several factors that should be considered when contextualizing our study with these results. In the study by Palou et al. [15], patients were treated with TURB, which was clinically judged complete by the surgeon, and did not undergo a second look TURB. Also in the study by Kluth et al. [16], data on second look TURB were not available. Despite the presence of detrusor muscle in all the specimens, which is considered a surrogate marker for the quality of the resection [22, 23], the residual disease could not be ruled out. Complete resection is essential for the optimal outcomes as it impacts prognosis and adjuvant therapy response in patients with NMIBC [24].

Both series could be biased by the relatively small number of patients and events. In a larger single-center retrospective series of 1021 patients treated with TURB and induction BCG, authors did not find an association of patients’ sex with disease recurrence or progression [25]. However, in this series, only 40% of the patients included had a T1 UBC.

We expanded upon these limitations by investigating the association of patients’ sex in a large multicenter dataset of T1 UBC. To the best of our knowledge, this is the largest series representing real-world clinical data. Moreover, our analyses adjusted relevant prognostic variables, which were not reported in other studies.

Another major limitation of the previously mentioned retrospective series is the administration of intravesical BCG therapy restricted to the induction course. Indeed, the efficacy of the treatment, particularly in patients with high-risk, is dependent on the maintenance schedule [26, 27]. We added detail on these shortcomings by investigating the association of patients’ sex with oncologic outcomes in patients adequately treated with BCG [17]. In this subgroup, the association of patients’ sex with disease progression could no longer be observed. This generates the hypothesis that an adequate BCG treatment might equalize the effect of patients’ sex on therapy response. However, the number of patients in this subgroup might have been too small to detect a statistically significant difference.

Our study is not devoid of limitations, which are mainly inherent to its retrospective design. During the large time span of study treatment modalities such as endoscopic image enhancement [28], changes in local clinical practices at each site and imaging modalities used for surveillance may have changed significantly. We had no information on the BCG strain used. However, there is no evidence on the differential effectiveness of one strain compared to another [29, 30], passaging and sub-culturing over the years may have changed the virulence of mycobacteria and immunological response in the host [31]. We had histopathology information at disease recurrence only for the first recurrence. Therefore, we could not assess whether the patient developed an HG-recurrence after a first low grade recurrence. Finally, we acknowledge the limitation of a missing central pathology review and the lack of information on histologic variants and lymphovascular invasion. Moreover, histopathological examination was performed according to institutional standards at each center, which could have led to heterogeneous results.

Despite all these limitations, our study has relevant clinical implications. Given the current world-wide BCG shortage, accurate selection of patients who are more likely to respond to the therapy is of paramount importance to avoid overtreatment, reduce complications, and drug wastage. We analyzed a cohort of NMIBC patients treated with BCG with the highest risk of disease recurrence and progression and found an association of female sex with disease progression. This evidence may help physicians during patient counselling regarding adjuvant therapies or early cystectomy and follow-up scheduling.

## Conclusion

Our analysis generates the hypothesis of differential oncologic outcomes in female compared to male patients if not adequately treated with BCG for T1G3/HG UBC. Further studies should focus on sex-based differences in innate and adaptive immune system and their association with BCG response. Moreover, these differences should be considered during trial planning for novel immunotherapy agents in NMIBC.

## Supplementary Information

Below is the link to the electronic supplementary material.

Supplementary file1 (DOCX 148 KB)
