# Supplementary material for: Association of patients’ sex with treatment outcomes after intravesical bacillus Calmette–Guérin immunotherapy for T1G3/HG bladder cancer
Source: World J Urol. 2021 Mar 13;39(9):3337–44. doi: 10.1007/s00345-021-03653-1 (PMC8510956; doi:10.1007/s00345-021-03653-1)
Supplement: Supplementary file 1 — Supplementary file1 (DOCX 148 KB) [file 345_2021_3653_MOESM1_ESM.docx]

**Supplementary figure S1.** Standardized mean difference of covariates before (blue line) and after (red line) inverse-probability weighting in 2635 patients treated with transurethral resection of the bladder (TURB) and adjuvant intravesical bacillus Calmette-Guérin (BCG) for T1G3/HG urinary bladder cancer
